# Choices and services related to contraception in the Gaza strip, Palestine: perceptions of service users and providers

**DOI:** 10.1186/s12905-019-0869-0

**Published:** 2019-12-19

**Authors:** Bettina Böttcher, Mysoon Abu-El-Noor, Nasser Abu-El-Noor

**Affiliations:** 1 Faculty of Medicine, Islamic University of Gaza, P. O. Box 108, Gaza, Gaza Strip Palestine; 2 Faculty of Nursing, Islamic University of Gaza, P. O. Box 108, Gaza, Gaza Strip Palestine

**Keywords:** Contraceptive choices, Contraceptive service, Male involvement in contraceptive use, Gaza strip, Palestine

## Abstract

**Background:**

Reliable contraception enables women and men to plan their family sizes and avoid unintended pregnancies, which can cause distress and anxiety, but also increase maternal mortality. This study explored potential barriers to contraceptive use for women in the Gaza Strip, Palestine from user and provider perspectives.

**Methods:**

A convenient sample was used to recruit women, who were current contraception users, from three healthcare clinics that provide family planning care, two governmental and one non-governmental. A 16-item questionnaire was completed by 204 women, including socio-demographic data, contraceptive use and eight questions exploring user experience. Additionally, 51 women attended focus groups for a deeper insight into their contraceptive use experience and potential barriers. Furthermore, 14 healthcare providers were interviewed about their experience with service provision. Quantitative data are presented as means and frequencies and qualitative data were analysed item by item and are presented in themes jointly with the quantitative data.

**Results:**

Women reported usage of only three main modern methods of contraception with 35.2% using intrauterine devices, 25.8% combined oral contraception and 16.4% condoms, while only 3.1% used the hormonal implant. Expectations from family planning services were low with most women attending the clinic having already decided their contraceptive method with decisions being made by husbands (41.2%) or women jointly with their partner (33.3%), only 13.7% took advice from service providers. Healthcare providers experienced high prevalence of beliefs that modern contraceptives cause infertility and cancer. Main barriers to effective family planning services were misconceptions of potential harm, poor availability and limited choice of contraceptive methods.

**Conclusion:**

Women’s contraceptive choices in Gaza are limited by prevalent misconceptions and fears as well as recurring shortages, negatively impacting fertility control. Men are a major factor in choosing a contraceptive method, however, they have limited access to information and therefore, potentially more misconceptions. Therefore, male community members need to be included in the delivery of information on contraceptives to increase women’s choice. Furthermore, greater access to long-acting reversible contraceptives, such as the hormonal implant, and improved availability might be key factors in improving contraceptive uptake in Gaza and, thus, reducing unintended pregnancies.

## Background

It has been estimated that the increase of contraceptive use over the last 20 years has reduced maternal mortality by 40% globally [1]. This is mainly achieved by reducing unintended pregnancies, increasing pregnancy spacing and reducing high risk pregnancies [2–5]. Therefore, contraceptive use improves health outcomes for women and their children [4, 6, 7]. However, contraceptive use prevalence remains variable among different countries ranging from 11.3% in Mozambique, 21.5% in Ghana, 54.0% in Bangladesh to 72.1% in Sweden [8–11]. The WHO included access to reliable contraception as a key strategy to achieve Sustainable Development Goals 3 (health and wellbeing) and 5 (gender equality) [12].

In Palestine, 54.8% of married women aged 15–49 years reported using contraception (herself or her partner), showing a slow but steady upward trend [13] and 44.0% of women of reproductive age used modern contraceptives [14]. Contraception is provided without charge to women of reproductive age from government and United Nations Relief and Works Agency (UNRWA) clinics. However, unintended pregnancies are not uncommon and cause distress as well as morbidity and mortality in women [15, 16]. Worldwide different factors were identified, that impact on women’s contraceptive usage, but limited data are so far available from Palestine [8, 17–21].

Therefore, this study explored factors that impact on the uptake of modern contraception in the Gaza Strip by investigating the experience and contraceptive practice of family planning (FP) service users, evaluating healthcare professionals’ perceptions of FP service provision and examining potential barriers to provide effective patient-centred care in this field.

## Methods

This is a cross-sectional study combining quantitative with qualitative approaches to data collection and thus providing in-depth data [22]. The use of both quantitative and qualitative approaches strengthens the design, reduces weaknesses in either approach and makes the study data more comprehensive [23, 24]. A convenience sample was used to recruit participants from three clinics providing FP in the Gaza Strip, two governmental clinics and one clinic run by a non-governmental organization (NGO) This clinic represents one of the largest NGO clinics providing SRH services and reaches patients from the entire Gaza Strip, who attend this clinic. Moreover, this clinic provides a greater choice than other clinics, including long- acting reversible contraception (LARC), such as hormonal implants. Contraceptive methods are provided free of charge or at a low cost. The government clinics are located in the same area, provding the usual SRH services to local women, which include the Medroxyprogesterone injecton and the intrauterince contraceptive device (IUD) as the only available LARC. Eligible for inclusion were all women of reproductive age who were at least 18 years old and who accessed sexual and reproductive health (SRH) services for family planning purposes for at least the second time. Excluded were women who attended for antenatal care services or who made their first visit. These same criteria were applied for recruitment of participants in the qualitative and the quantitative parts of the study. Furthermore, all healthcare professionals from the three healthcare facilities, who had worked at least 1 year in the setting, were interviewed.

### Quantitative data collection

The quantitative data were collected with a self-administered questionnaire in Arabic, consisting of a total of 16 items covering socio-demographic details as well as experience of FP services received and actual contraceptive use (see Additional file 1). Experience of sexual and reproductive health services was scored on a four-point scale with 1 = strongly disagree, 2 = moderately disagree, 3 = moderately agree and 4 = strongly agree for each item. Prior to using the instrument, it was pilot-tested on 15 service users in the centres to test clarity and user-friendliness of the instrument, which were not included in the study.

### Qualitative data collection

In total, five focus groups were conducted consisting of 8–12 women, who had accessed SRH services. Three focus groups took place at the NGO clinic and one at each of the two government clinics. Focus groups were held in a private room of the respective healthcare facility with one female researcher as facilitator. After explanation of the purpose of the study and obtaining a written consent from each participant, questions were posed to the group to stimulate a discussion and sharing of views on each topic. Contributions were solicited from all participants, although not all necessarily contributed to each question (see Additional file 2). The sessions were audio-recorded and later transcribed verbatim, including each individual question and all contributions. Healthcare professionals were recruited to participate in semi-structured, private interviews conducted by one of the researchers (see Additional file 3). The interviews were audiotaped and transcribed verbatim.

### Data analysis

Data from the quantitative arm of the study were analysed with the Statistical Package for Social Sciences (SPSS) version 18 (IBM, Chicago, Illinois) and are presented as means (±standard deviations), percentages and frequencies. Cronbach α was 0.771, showing good reliability of the questionnaire. Furthermore, the independent sample t-test was used to test differences between views by participants from government and NGO clinics. A *p*-value of ≤0.05 was regarded as significant.

Data from the focus groups and interviews with healthcare professionals were analysed through careful reading of the responses by each researcher followed by identifying, coding and categorizing of data and using thematic analysis, to process the qualitative information [25]. Throughout data coding, the research team extracted themes and gave an appropriate label for each. Some quotes from the participants are presented to provide comprehensive views of these themes.

### Ethical considerations

Prior to data collection via questionnaires, focus groups or interviews, the purpose of the study was explained to potential participants, it was explained that participation was entirely voluntary, had no impact on health services received or employment status and could be terminated at any time throughout the study. Written consent was obtained from all participants. All data were kept anonymously and an agreement of complete confidentiality was made in every focus group.

## Results

A total of 213 women completed the questionnaires, of which nine were excluded because more than three values were missing from their questionnaires, leaving 204 participants, including 123 (60.0%) from the NGO clinic as well as 47 (22.9%) and 34 (16.6%) from the two government clinics respectively. From these 158 (77.6%) women were current contraceptive users and gave information on their current use. Altogether, five focus groups were conducted with a total of 51 participants, 35 in the NGO clinic and eight in each of the government clinics. Furthermore, 14 healthcare providers were interviewed from all clinics.

### Characteristics of participants

The mean age of survey participants was 30.8 (±7.5) years; ranging from 19 to 50 years. The vast majority of 180 participants (88.2%) had benefitted from secondary school or university education. However, the majority were housewives, with low household incomes (Table 1). The mean age of women taking part in the focus groups was 30.1 (±6.2) years with 36 women (70.6%), having benefitted from education of secondary school level or above (Table 1). Most participants were housewives, although many expressed a wish to work, but only three (5.9%) were in formal employment, all of whom attended the same governmental clinic. Monthly household income varied greatly from 0 to $675, but was generally poor (Table 1). In total, 154 women (60.4%) reported incomes below $300 per month.

**Table 1:** Sociodemographic characteristics of study participants

	Quantitative study arm participants *n* = 204			Qualitative study arm participants *n* = 51		
Variable	Mean ± SD	Minimum	Maximum	Mean ± SD	Minimum	Maximum
Age	30.8 (±7.5)	19	50	30.1 (±6.2)	21	48
Income (in American Dollars)	$304.5 (± $257.5)	0	$725	$182.9 (± $156.4)	0	$675.0
Education	Total numbers	Percentage		Total numbers	Percentage	
Primary school and below	4	2.0%		3	5.9%	
Preparatory school	17	8.3%		8	15.7%	
Secondary school	94	46.1%		15	29.4%	
University degree	86	42.2%		21	41.2%	
Unknown	3	1.5%		4	7.8%	

### Perceptions of women using family planning services

#### Quantitative data analysis

Overall assessment of FP services showed similar trends in both NGO and government clinics, although overall assessments were more positive in the NGO clinic. In both groups, the most positive evaluation was given for ‘Written instructions are in a clear and understandable language’ with a mean of 3.59 and 3.15 (from the maximum score of 4) respectively. Similarly, the most negative response was gained on the same item, which was ‘I received adequate information about contraceptive choices, including advantages and disadvantages of each method’ with 3.30 and 2.66. The range between most positive and most negative response was greater in the government clinics than in the NGO (Table 2). All differences were statistically significant with p = < 0.01. Participants in the NGO clinic gave significantly higher mean scores in all categories, compared to those in the government clinics (*p* value < 0.01). However, no significant differences were found between the two government clinics. These are therefore, presented as one total in Table 2.

**Table 2:** Satisfaction of participants with different aspects of family planning services

	Variable 1 = strongly disagree, 2 = moderately disagree, 3 = moderately agree and 4 = strongly agree	Mean (±Standard deviation)			*P* value
		Total n = 204	NGO Clinic *n* = 123	Goverment Clinics *n* = 81	
1.	I received adequate information about contraceptive choices, including advantages and disadvantages of each method.	3.08 (±0.94)	3.30 (±0.79)	2.66 (±1.05)	< 0.01
2.	I gained a good understanding of contraceptive methods from the explanation given to me in the clinic.	3.16 (±0.82)	3.40 (±0.63)	2.74 (±0.96)	< 0.01
3.	Contraception is always available, including the required oral contraception	3.22 (±0.82)	3.40 (±0.65)	2.89 (±0.99)	< 0.01
4.	I was always given full freedom to choose the contraception I preferred.	3.36 (±0.75)	3.49 (±0.64)	3.12 (±0.87)	< 0.01
5.	Healthcare professionals were supportive of my choice.	3.36 (±0.76)	3.52 (±0.67)	3.07 (±0.84)	< 0.01
6.	Educational materials (such as brochures) are available at this health care center and help in my understanding of contraceptive choices.	3.21 (±0.78)	3.46 (±0.60)	2.73 (±0.87)	< 0.01
7.	Staff make sure that I understand their instructions.	3.28 (±0.72)	3.43 (±0.58)	2.97 (±0.86)	< 0.01
8.	Written instructions are in a clear, understandable language.	3.44 (±0.73)	3.59 (±0.56)	3.15 (±0.92)	< 0.01

#### Qualitative data analysis

The main themes extracted from the qualitative data analysis were a general good level of satisfaction about the services in all clinics, with more positive views in the NGO clinic, when compared to government clinics. Most women went to the clinic with a certain method in mind, they felt the Staff were supportive and answered all questions well. Participants acknowledged a high prevalence of disparate, possibly misleading, information in the community.

Focus group participants at the NGO clinic experienced FP services as good and comprehensive. The Staff was described to be knowledgeable and professional, dealing equally well with feto-maternal complications or preeclampsia as with psychosocial difficulties. Participants described a holistic approach, which addressed the individual woman in the context of their family situation, which was appreciated by the participants and expressed as follows:

*‘The fetus had a heart problem. They told me that there was a hole in his heart. They have a good ultrasound that helped in the diagnosis of this case’.*

*‘I used to be classified as a high-risk pregnancy. I have pregnancy diabetes and hypertension. Every time I go to the UNRWA clinic, they refer me to the hospital. When I came to the NGO clinic, they took care of me and made me feel that my case is not that dangerous. This gave me reassurance and I continued the pregnancy and gave birth safely.’*

‘*They talk to me and cheer me up. It is not only about medical treatment.’*

Participants in the government clinics also reported general satisfaction with services and it was clear that the participants’ expectations were met. Although some found waiting times long, consultations brief and lacking detailed information about contraceptive choices, all agreed that questions were answered precisely. Participants reported to have heard many opinions about contraception, usually from relatives and friends, and often attended the FP clinics with a certain method in mind they wanted to use, not expecting to gain information from healthcare professionals, but to be given what they asked for at the consultation. This is reflected also in the mean scores of 2.66 and 2.74 (Table 2) for the two associated items, indicating most women were satisfied with the information they received. Advice given to women was reported to have impact on their contraceptive choice, by some women, while most decided without healthcare providers’ input.

*‘I came to take contraceptive pills. Any question I ask they answer.*’

*‘They explained the disadvantages about IUD only, but didn’t explain all advantages and disadvantages of other methods, but the information was relatively enough. I changed my mind and selected pills.*’

‘*They don’t explain anything. I came here and asked for pills. They gave them to me without providing me any further explanation.’*

The highest score in both clinics was given for clarity of written information. In the NGO clinic this was supported also by a video that was delivering information while women were waiting in the waiting room and accessible staff to ask questions. This informal delivery of information was found to be helpful by participants. Participants in the NGO clinic said:

*‘I heard so many things about contraception that I did not know what was true and what was wrong. Here they give you good advice. I trust them.’*

*‘They give me complete information about contraceptives from A to Z.’*

Although the question on the clarity of written information received the highest score in the government clinic with 3.15 of a maximum 4, focus group participants reported that they did not receive any written information. However, the research team saw pamphlets about contraception in the clinic, possibly reflecting inconsistency in their distribution to women.

### Contraceptive choice

Participants reported, and the actual use of contraception demonstrated, a limited choice of contraception for the women of the Gaza Strip (Table 3). The most commonly used contraceptives were the intrauterine device (IUD), used by 56 (35.4%) women, and combined oral contraceptives (COCP) by 41 (25.9%), followed by less reliable choices, namely condoms with 26 women (16.5%) and natural methods with 24 (15.2%). Although introduced to the NGO clinic as a novel choice 2 years prior to this study, only five women (3.2%) reported to be using the hormonal implant for contraception at the time of data collection. Participants reported that they were free to choose the method of contraception and that they were supported in this by staff with positive responses for both items in the questionnaire with 3.36 out of 4.0 for both items (Table 2). When asked who mostly influenced their choice of contraception, most focus group participants (*n* = 21; 41.2%) answered their husbands, 17 (33.3%) women decided together with their husbands and a minority of only six (11.8%) women reported to decide by themselves alone. Seven women (13.7%) also took the advice of healthcare staff to guide their choice.

**Table 3:** Contraceptive use in total numbers and (percent)

Type of contraception	Total	Government	NGO clinic
IUD	56 (35.4%)	22 (32.4%)	34 (37.4%)
Oral contraceptives	41 (25.9%)	22 (32.4%)	19 (20.9%)
Condom	26 (16.5%)	13 (19.1%)	13 (14.3%)
Natural methods	24 (15.2%)	9 (13.2%)	16 (17.6%)
Implant	5 (3.2%)	0 (0.0%)	5 (5.5%)
Injections	4 (2.5%)	0 (0.0%)	4 (4.4%)
Other methods	2 (1.3%)	2 (2.9%)	0 (0.0%)
Total	158	68	91

### Perceptions of family planning service delivery by healthcare providers

Healthcare professionals reported several barriers in the delivery of FP services to women in the Gaza Strip, which were present in both, NGO and government clinics. A big challenge described by healthcare professionals was the high prevalence of misconceptions about contraception in the community, including women attending all three healthcare facilities. The most common misconceptions were that contraceptive methods had a negative impact on future fertility as well as an overestimation of their association with cancer among females, especially breast cancer. Healthcare professionals found re-educating women on these issues was challenging.

*‘Some challenges included the traditions of the community and their attitude toward family planning. It was hard to change their attitude. Let's say not to change them, but let’s say to correct them and change or correct some behaviors. Always changing behavior is difficult. It takes a lot of effort from the team. It is difficult to change these misconceptions.’*

Significant shortages of contraception have to be overcome regularly, requiring women to change their contraceptive method. Some women in the focus groups also reported this problem, but for healthcare professionals it was an even bigger challenge. Women were also referred from other clinics, if methods were unavailable at one place.

‘*The contraceptive methods are not available all the time,’*

*‘Last year progesterone only pills were not available for eight months of the year*.’

*‘At UNRWA clinics, they serve a large number of women. When they are out of contraceptives, they send them to us* [the NGO clinic].*’*

Generally, it was felt by healthcare staff that providing reliable contraception is not regarded as a priority in service provision by the Palestinian Ministry of Health (MoH), due to the high pressure on the service. But the staff in all three facilities reported working hard to provide adequate FP services.

*‘Family planning is continuous, we offer this service throughout the year and even during the war. It is very essential in such hard economic conditions as in the Gaza Strip’*

## Discussion

Women in governmental and NGO clinics made similar experiences when accessing FP services in terms of positive and negative points, but the NGO setting gained consistently more positive assessments in all areas by its service users. The main barriers for women to use contraception were the beliefs that their use might lead to infertility or cancer, as well as shortages of some contraception, the latter leading to limited choice of contraception for women in the Gaza Strip. Consequently, the most commonly used methods were the IUD, COCP and condoms with very few women using other modern methods.

### Contraceptive choice

The expectations that women had of the consultation in terms of information delivery about contraceptive methods were low and those were mostly satisfied. Most information on contraception was received informally in the community, which might be one important factor for the limited choice that women made regarding contraception with only three main methods used. Surprisingly low was the use of long-acting reversible contraception (LARC) in this study with only the IUD being used from this category, which was similar to findings by Hababeh et al., who surveyed Palestinian refugee mothers attending UNRWA clinics [26]. Most women receive contraception in the UNRWA or government clinics, which do only offer the Medroxyprogesterone injection and the IUD as LARC. But even in the NGO clinic, which introduced the hormonal implant as another choice, uptake was very poor, which might be due to limited awareness of this method within the community. In concordance with this study, the COCP remains the most widely used contraception worldwide, as confirmed in Sweden, the USA, Saudi Arabia, Bangladesh and Mozambique [8, 21, 27–29]. However, globally a greater choice of contraception has become available to women and a steady increase in LARC use has been seen [30, 31], while in the Gaza Strip no change in the used methods has occurred over the last years with developments and advances in contraception. This indicates an acute limitation of contraceptive choice for women in the Gaza Strip [32], leading to unintended pregnancies as well as increased maternal mortality rates, mostly by causing unintended pregnancies in women with comorbidities [15, 31, 32]. Furthermore, this limited choice puts women and their families at an acute disadvantage, as they cannot achieve their chosen family sizes, leading to less safe attempts of termination of pregnancies in some cases [15]. LARC has been proven to meet women’s contraceptive needs more reliably and achieving greater satisfaction among them [33], even in those women who did not set out to use LARC. Germain et al. suggested that availability of five different methods of contraception, including hormonal and non-hormonal, as well as short-term and long-term methods, to be the minimum requirement for acceptable quality FP services [33]. Although this is mostly achieved in Gaza (interrupted by recurring periods of acute shortages), only three modern methods (IUD, COCP and male condoms) were actually used. Reasons for this might be that women do not get information on contraception from FP providers, but rather from their community as well as the fact that these three methods are available consistently and free of charge at government and UNRWA clinics. Consequently, FP providers reported that influencing women’s choice was extremely difficult.

### Barriers to use of modern contraceptives

The main barriers to the use of contraceptives by participants of this study, Palestinian women living in the Gaza Strip, were misconceptions about contraceptives, poor availability and limited choice. Erroneous beliefs about modern contraceptives mainly included fear of them causing infertility and cancer. Such misconceptions were also found in other studies from Saudi Arabia [21], Nigeria [19], Tanzania [34], Sweden [11], the USA [20] and a global study on the IUS [35].. Therefore, such beliefs are globally prevalent despite the long history of modern contraceptive use and the funds that have gone into global education on contraception. One factor contributing to this might be the decision-making process reported by women with only 13.7% of participants in this study taking the advice of healthcare professionals, while the majority leaves their husbands to decide or makes the decisions together with their partners. However, husbands usually do not attend the SRH clinics with their wives and therefore do not have the same access to accurate information about contraception. Their level of erroneous beliefs might be greater and at the same time have larger influence on the decision to use contraception; a trend also seen in other global studies [18, 20, 36–38]. On the other hand, male support for contraceptive use increased female uptake [39–41]. Therefore, male partners have to be included in FP service delivery and education on contraception has to be delivered to the entire community for effective communication about contraceptive methods, their benefits and risks. Targeting specific groups with information on LARC has been shown to be effective, such as among college students in the USA [42] and appropriate campaign content can be devised [29], which could include male community members. Therefore, highest impact on increasing uptake of modern contraception can be achieved in low-resource settings by providing a greater choice of contraceptive methods in combination with commitment to include the wider community, as well as male community members in FP education..

### Strengths and limitations

The mixed approach used in this study, as well as the inclusion of service users and providers, allowed an in-depth view of experiences by family planning service users and providers, their contraceptive uptake and challenges in the Gaza Strip. The setting of focus groups and interviews created an environment of comfort for women and providers to share their experiences beyond the quantitative outcomes. However, data collection was limited to women already attending FP services, leading to selection bias and limiting generalizability of findings. Furthermore, this being a convenient sample, no prior power calculation had been made. Moreover, the sample size was small compared to the total of reproductive age women in the Gaza Strip and their contraceptive needs, further limiting generalizability of results. The strength of this study is not representing the views of all reproductive age women in the Gaza Strip or Palestine, but offering a closer look at a neglected area of the health sector in Gaza, highlighting the challenge for women to secure appropriate contraceptive choices for themselves.

## Conclusion

Tackling misconceptions within the community on contraception causing harm, as well as ensuring greater choice and consistent availability of contraception will transform the opportunities for Gazan women to control their fertility. Male involvement in FP education and community engagement in FP services are the main avenues to reach such goals. Increasing knowledge and availability of LARC, such as the hormonal implant, might be a key policy for improving contraceptive choice and uptake in Gaza and, thus, reducing unintended pregnancies. FP services need higher local priority in order to achieve implementation of such policies.

## Supplementary information

**Additional file 1.** The 16-item questionnaire used for quantitative data collection.

**Additional file 2.** List of questions put to the focus groups for discussion.

**Additional file 3.** List of questions put to healthcare professionals for discussion in semi-structured interviews.

## Data Availability

The datasets used and analysed during the current study are available from the corresponding author on reasonable request.

## Abbreviations

COCP: Combined oral contraceptive pill
FP: Family planning
IUD: Intrauterine contraceptive device
LARC: Long-acting reversible contraception
MoH: Ministry of Health
NGO: Non-governmental organisation
SRH: Sexual and reproductive health services
UK: United Kingdom
UNRWA: United Nations relief and works agency
USA / US: United States of America
WHO: World Health Organization
