# Supplementary material for: Choices and services related to contraception in the Gaza strip, Palestine: perceptions of service users and providers
Source: BMC Womens Health. 2019 Dec 19;19:165. doi: 10.1186/s12905-019-0869-0 (PMC6923918; doi:10.1186/s12905-019-0869-0)
Supplement: Supplementary file 1 — Additional file 1. The 16-item questionnaire used for quantitative data collection. [file 12905_2019_869_MOESM1_ESM.docx]

**Additional file 1**

**Patient Questionnaire**

Dear participant

We are a research team from the Islamic University of Gaza, wanting to assess how women use sexual and reproductive health services, what their contraceptive usage is and if there are any weaknesses in the provision of current sexual and reproductive health services. The aim of this study is to explore any difficulties for women to access and benefit from family planning services. Furthermore, we want to look at current use of contraception and potential ways of improving access and use of family planning services in Gaza.

Your participation is voluntary. You have the right to refuse in participation, or to refuse to answer any question in the list. If you refuse to be included in this study, the services you receive in this center will not be affected. Participation in this study will not pause any physical, psychological or emotional harm nor it will have any financial burden on you.

We also would like to assure you that you don't need to write your name on the questionnaire. When we will report about the results, no names will be included in the report.

If you agree to be involved in this evaluation process, you are kindly asked to fill in this questionnaire. Filling in this questionnaire is considered as a consent that you agree to be involved in the evaluation of this program.

Responding to the items of this questionnaire takes only about 10-15 minutes. Upon completion of the questionnaire, please give it back to me. If you feel like you need further explanation about any item of the questionnaire, please don't hesitate to ask for clarification.

Date: ______________________ Signature;____________________________

**Part 1:**

1. Name of the health care center:  Rimal Government Clinic, Gaza  Government clinic, Dier Elbalah ,  Women Health Centre, Al-Bureij
2. What is your age in years: ______________
3. Where do you live: __________________________
4. What is the highest level of your education: _______________________
5. What is your monthly household income:__________________
6. What is the type of reproductive health services you receive now?
   1. Antenatal
   2. Family Planning
   3. Sexual and Reproductive Health Advice
   4. Postnatal
   5. Others:-------------------------------specify ……………………………………….
7. For how long are you receiving Reproductive Health Services in this center _____________________________________

**Part 2**:

| 1. Do you receive family planning service now (or within the last year) at this center   Yes  If the answer was yes, please continue answering the rest of the questions  No  If the answer is no; you are finished. | | | | |
| --- | --- | --- | --- | --- |
| 1. Which contraception do you use now? Please circle: 2. Oral contraceptive pill B) IUCD   C) Implant D) Condom  E) Injections F) Natural methods  G) Other: please specify:__________________________________________ | | | | |
| 1. I received adequate information about contraceptive choices, including advantages and disadvantages of each method. | 1 | 2 | 3 | 4 |
| 1. I gained a good understanding of contraceptive methods from the explanation   given to me in the clinic. | 1 | 2 | 3 | 4 |
| 1. Contraception is always available, including the required oral contraception. | 1 | 2 | 3 | 4 |
| 1. I was always given full freedom to choose the contraception I preferred. | 1 | 2 | 3 | 4 |
| 1. Healthcare professionals were supportive of my choice. | 1 | 2 | 3 | 4 |
| 1. Educational materials (such as brochures) are available at this health care center   and help in my understanding of contraceptive choices. | 1 | 2 | 3 | 4 |
| 1. Staff make sure that I understand their instructions. | 1 | 2 | 3 | 4 |
| 1. Written instructions are in a clear, understandable language. | 1 | 2 | 3 | 4 |
