# Supplementary material for: Choices and services related to contraception in the Gaza strip, Palestine: perceptions of service users and providers
Source: BMC Womens Health. 2019 Dec 19;19:165. doi: 10.1186/s12905-019-0869-0 (PMC6923918; doi:10.1186/s12905-019-0869-0)
Supplement: Supplementary file 2 — Additional file 2. List of questions put to the focus groups for discussion. [file 12905_2019_869_MOESM2_ESM.docx]

**Additional file 2**

**Sample of questions to be used with Focus Groups for the clients**

**Talk to us about your experience during receiving reproductive health services in this center!**

**حدثينا عن تجربتك بخصوص تلقي خدمات الصحة الانجابية في هذا المركز الصحي**

1. How was the staff’s attitude towards you?

كيف تقيمين تعامل أعضاء الفريق الصحي معك؟

1. What are the major advantages you found related to the services you received in this center?

ما هي المزايا الرئيسية التي وجدتها ذات الصلة بالخدمات الصحية الانجابية التي تلقيتيها في هذا المركز؟

1. What are the major disadvantages you found related to the services you received in this center?

ما هي العيوب الرئيسية التي وجدتها ذات الصلة بالخدمات الصحية الانجابية التي تلقيتيها في هذا المركز ؟

1. How do you think this service met your reproductive health needs or resolved your health concerns?

كيف ترين أن هذه الخدمات الصحية الانجابية نجحت في حل مشاكلك و اهتماماتك الصحية ذات الصلة؟

1. How do you think were your family planning needs met?

إلى أي حد تعتقدين أنه تم تلبية احتياجاتك المتعلقة بتنظيم الأسرة؟

1. How was the explanation of different contraceptive methods? What was included?

كيف كان الشرح عن وسائل منع الحمل المختلفة؟ ما الذي تم تضمنته؟

- 1. Disadvantages / Advantage

المميزات و العيوب

- 1. Possible side effects?

الآثار الجانبية المحتملة

1. How much time was given for such explanation and counselling?

كم من الوقت تم تخصيصه للشرح و اعطاء المشورة

1. How was the staff responding to your questions?

كيف كانت استجابة الفريق الصحي لأسئلتك؟

1. How did the explanation influence your choice?

كيف أثرذلك على اختياراتك (قراراتك) لوسيلة منع الحمل؟

1. How helpful was any written information given to you?

إلى أي درجة ساعدتك المعلومات الصحية المكتوبة (منشورات, مطويات)

1. How did you make your choice of contraception?

كيف كان اتخاذ القرار باستخدام وسائل منع الحمل؟

- 1. What had the biggest influence on your choice of contraception?

ما هو العامل الأكثر تأثيرا في اتخاذ القرار باستخدام وسائل منع الحمل؟

- 1. Who had the biggest influence on your choice of contraception?

من هو الشخص الأكثر تأثيرا في قرار استخدام منع الحمل؟

1. What would prevent you from receiving reproductive health services provided by this center?

ما هي العوامل التي من الممكن أن تمنع أو تعيق تلقيك جميع خدمات الصحة الإنجابية التي يقدمها هذا المركز؟

1. What do you recommend to improve the reproductive health services in this center?

ما الذي توصين به لتحسين خدمات الصحة الإنجابية في هذا المركز؟

1. Would you recommend these services to your friends or relatives?

هل ستوصي أصدقائك وأقاربك بالاستفادة من هذه الخدمات؟
