# Supplementary material for: Choices and services related to contraception in the Gaza strip, Palestine: perceptions of service users and providers
Source: BMC Womens Health. 2019 Dec 19;19:165. doi: 10.1186/s12905-019-0869-0 (PMC6923918; doi:10.1186/s12905-019-0869-0)
Supplement: Supplementary file 3 — Additional file 3. List of questions put to healthcare professionals for discussion in semi-structured interviews. [file 12905_2019_869_MOESM3_ESM.docx]

**Sample of questions to be used in the semi-structured interviews with the health care providers**

**Talk to us about your experience during providing the sexual and reproductive health services in this center!**

1. What are the major advantages you found related to the services you provide in this center?
2. Do you feel competent in counselling women on contraceptive advice?
3. What are the major obstacles/barriers you face during delivering services to you clients in this center?
4. What are the main difficulties you face in giving advice on contraceptive methods?
5. How do you think this service meets the sexual & reproductive health needs of your clients?
6. What do you recommend to improve the sexual & reproductive health services in this center?
7. What other services associated with sexual & reproductive health do you recommend to be added in this center?
8. How do you evaluate these services?
